# Longitudinal associations of naturalistic psychedelic use with psychotic and manic symptoms

**DOI:** 10.1017/S0033291725000716

**Published:** 2025-03-31

**Authors:** Otto Simonsson, Simon B. Goldberg, Walter Osika, Cecilia U. D. Stenfors, Sankalp Chaturvedi, Caroline M. Swords, Jayanth Narayanan, Peter S. Hendricks

**Affiliations:** 1 Department of Neurobiology, Care Sciences and Society, Karolinska Institutet, Stockholm, Sweden; 2 Center for Healthy Minds, University of Wisconsin – Madison, Madison, WI, USA; 3 Department of Counseling Psychology, University of Wisconsin – Madison, Madison, WI, USA; 4 Department of Psychology, Stockholm University, Stockholm, Sweden; 5 Department of Management and Entrepreneurship, Imperial College London, London, UK; 6 Department of Management & Organizational Development, Northeastern University, Boston, MA, USA; 7 Department of Psychiatry and Behavioral Neurobiology, University of Alabama at Birmingham, Birmingham, AL, USA

**Keywords:** bipolar, illegal drug use, mania, psychedelics, psychosis, schizophrenia

## Abstract

**Background:**

Findings from contemporary clinical trials suggest that psychedelics are generally safe and may be effective in the treatment of various psychiatric disorders. However, less is known about the risks associated with psychedelic use outside of medically supervised contexts, particularly in populations that are typically excluded from participation in clinical trials.

**Methods:**

Using a preregistered longitudinal observational research design with a purposive sample of US residents between 18 and 50 years old (*N*=21,990), we investigated associations between self-reported naturalistic psychedelic use and psychotic and manic symptoms, with emphasis on those with psychiatric histories of schizophrenia or bipolar I disorder.

**Results:**

The follow-up survey was completed by 12,345 participants (56% retention), with 505 participants reporting psychedelic use during the 2-month study period. In covariate-adjusted regression models, psychedelic use during the study period was associated with increases in the severity of psychotic and manic symptoms. However, such increases were only observed for those who reported psychedelic use in an illegal context. While increases in the severity of psychotic symptoms appeared to depend on the frequency of use and the intensity of challenging psychedelic experiences, increases in the severity of manic symptoms appeared to be moderated by a personal history of schizophrenia or bipolar I disorder and the subjective experience of insight during a psychedelic experience.

**Conclusions:**

The findings suggest that naturalistic psychedelic use specifically in illegal contexts may lead to increases in the severity of psychotic and manic symptoms. Such increases may depend on the frequency of use, the acute subjective psychedelic experience, and psychiatric history.

## Introduction

There has been a resurgence of scientific interest in psychedelics as an adjunct to psychotherapy (i.e. psychedelic-assisted therapy) for treating various mental health conditions (Nutt & Carhart-Harris, 2021). For example, recent studies indicate that psychedelic-assisted therapy is associated with reductions in depressive symptoms in patients with major depressive disorder or treatment-resistant depression (Goodwin et al., 2022; Raison et al., 2023) and reductions in anxiety symptoms in patients with life-threatening cancer (Griffiths et al., 2016; Ross et al., 2016). The evidence from modern-day trials suggests that psychedelics are relatively safe in controlled settings and in some specific psychiatric populations (Roscoe & Lozy, 2022; Simonsson et al., 2023a), but less is known about risks associated with psychedelic use outside of tightly controlled clinical trials (e.g. Evans et al., 2023; Simonsson, Johnson, & Hendricks, 2024a), especially in potentially vulnerable populations.

Modern clinical trials usually implement exclusion criteria that disqualify individuals with either a personal or family history (e.g. first- or second-degree) history of psychotic or bipolar disorders (Johnson, Richards, & Griffiths, 2008). These criteria partially exist based on the concern that psychedelic use may trigger or worsen psychotic or manic symptoms in these populations (Strassman, 1984). Notably, however, in a recent clinical trial using psychedelic-assisted therapy in patients with bipolar II disorder, no increases in psychotic or manic symptoms were reported (Aaronson et al., 2024). Yet little is known about the risk of psychotic or manic episodes following naturalistic psychedelic use that lacks the extensive safeguards of a clinical trial, especially among individuals with other psychiatric histories of concern such as schizophrenia or bipolar I disorder (Barber, Nemeroff, & Siegel, 2022; Bosch, Halm, & Seifritz, 2022; Dos Santos, Bouso, & Hallak, 2017; Gard et al., 2021; Johnson et al., 2008; Sapienza et al., 2023, 2025; Wolf et al., 2023). Such epidemiological research has become increasingly urgent in countries such as the United States, where there have been rapid increases in naturalistic psychedelic use (Walsh, Gorfinkel, Shmulewitz, Stohl, & Hasin, 2024), as well as recent legislative reforms to legalize or decriminalize the use of certain psychedelics in some jurisdictions (Siegel, Daily, Perry, & Nicol, 2023).

Recent epidemiological research has sought to quantify the risk of psychotic or manic episodes following naturalistic psychedelic use among individuals with a history of psychotic or bipolar disorders (Honk et al., 2024; Morton et al., 2023; Simonsson et al., 2023b, 2024b). For example, in a recent cross-sectional study, among adolescents with a greater genetic vulnerability to schizophrenia or bipolar I disorder, naturalistic psychedelic use was associated with more manic symptoms than in individuals with a lower genetic vulnerability (Simonsson, Mosing, et al., 2024b). Another recent study using a longitudinal research design found that naturalistic psychedelic use during the study period was associated with a decrease in the number of psychotic symptoms among participants with a personal history of psychotic disorders and an increase in the number of psychotic symptoms among those with a personal history of bipolar disorders. However, the study did not ask participants to report manic symptoms or specify the type of psychotic or bipolar disorder in their personal history (Honk et al., 2024), which precluded exploration of disorder-specific interactions with naturalistic psychedelic use on psychotic or manic symptoms. Prior work has also not investigated the quality of the acute psychedelic experience (e.g. psychologically challenging experiences, insightful experiences) and its association with psychotic or manic symptoms.

Since individuals with a personal or family history of psychotic or bipolar disorders are typically excluded from participation in clinical trials involving psychedelics, exploring naturalistic psychedelic use offers an opportunity to investigate psychiatric risks in these populations. Using a longitudinal research design with a large US sample (*N*=21,990), we conducted a study to better understand associations between naturalistic psychedelic use and psychotic or manic symptoms, with a specific focus on individuals with histories of certain psychotic or bipolar disorders. We aimed to longitudinally investigate (1) associations between naturalistic psychedelic use and changes in the severity of psychotic or manic symptoms; and (2) interactions between naturalistic psychedelic use and specific psychiatric histories (i.e. schizophrenia, bipolar I disorder) on changes in the severity of psychotic or manic symptoms.

## Methods

### Participants

The participants in this study were recruited through Prolific Academic (https://prolific.com), which is an online platform that facilitates study participant recruitment for researchers. Using purposive sampling to maximize the number of participants who were likely to report psychedelic use during the study period, we recruited US residents between 18 and 50 years old, based on results from a recent longitudinal study showing that 83% of participants who reported psychedelic use during the study period were 47 years or younger (Simonsson et al., 2023d). We initially aimed to recruit at least 13,000 participants between 18 and 50 years of age, which we estimated would result in at least 395 participants who would report psychedelic use during the study period. However, only 336 participants reported psychedelic use during the first study period in 2023. We therefore continued recruitment during 2024 and aimed to recruit at least 7000 additional participants. The study (design plan, sampling plan, variables, and analysis plan) was preregistered and updated on the Open Science Framework (OSF) at https://osf.io/249db

### Materials and procedure

Participants were recruited from June 2023 until September 2023 and in June 2024. Participants completed a baseline survey and were invited to complete a follow-up survey approximately two months later. This study was part of a larger survey and participants received a small monetary reimbursement upon completing both the baseline and follow-up surveys. Study procedures were determined to be exempt from review by the Institutional Review Board at the University of Wisconsin–Madison.

### Measures

#### Psychedelic use

All participants were asked at baseline to report which, if any, of the following psychedelics they had used in the past two months: psilocybin, ayahuasca, N, N-Dimethyltryptamine (DMT), lysergic acid diethylamide (LSD), mescaline, peyote, or San Pedro. At follow-up, participants were asked to report which, if any, of the same psychedelics they had used during the two-month study period. Those who reported psychedelic use at follow-up were asked how many times they used psychedelics in the past two months (once, twice, three times or more). The same participants were asked to recall their most intense psychedelic experience during the study period and complete the Challenging Experiences Questionnaire (CEQ; Barrett, Bradstreet, Leoutsakos, Johnson, & Griffiths, 2016) and the Psychological Insight Questionnaire (PIQ; Davis et al., 2021), which were developed to assess psychologically difficult states and the subjective experience of insight, respectively, during psychedelic experiences. The responses for CEQ were rated on a 0- (“‘None; not at all”) to 5-point (“Extreme”) Likert scale and the responses for PIQ were rated on a 0- (“No, not at all”) to 5-point (“Extremely [more than ever before in my life]”) Likert scale. The internal consistencies for CEQ and PIQ in the current sample were excellent (Cronbach’s alpha = 0.98 and 0.97, respectively). The same participants were also asked to report the type of psychedelic and the dose they used (i.e. low, moderate, large, very large, extremely large) for their most intense psychedelic experience during the study period, identify any applicable extra-pharmacological factors of interest (e.g. lack of psychological support; list from Simonsson, Hendricks, Chambers, Osika, & Goldberg, 2023c), indicate the date of the experience, and specify the city closest to where they had the experience. Depending on the laws and regulations applicable to the specified city at the date of their most intense psychedelic experience during the study period, we constructed a legal status variable with three levels (0 = no psychedelic use, 1 = psychedelic use in illegal context, 2 = psychedelic use in non-illegal [i.e. decriminalized or legal] context).

#### Demographics and other substance use

At baseline, all participants were asked to report their age (in years), gender identity, educational attainment, degree of religiosity, and political affiliation (see Supplemental Materials for survey items on demographics). At follow-up, participants were also asked to report past two-month use (yes, no) of alcohol, nicotine products (e.g. cigarettes, e-cigarettes, cigarillos, little cigars, and smokeless tobacco), cannabis products (e.g. weed, tetrahydrocannabinol (THC), cannabidiol (CBD), hemp oil), 3,4-methylenedioxymethamphetamine (MDMA), major stimulants (e.g. cocaine, methamphetamine), illicit narcotic analgesics/opioids (e.g. morphine, heroin, and oxycodone), illicit benzodiazepines and barbiturates (e.g. Diazepam [Valium], Alprazolam [Xanax]), inhalants (poppers, whip-its, nitrous oxide, and glue), and other substances.

#### Psychiatric history

At baseline, all participants were asked to report their personal psychiatric history. Specifically, participants were asked if they had a current or past diagnosis of any of the major diagnostic classes listed in the DSM-5-TR (see Supplemental Materials for full list). If participants reported a personal history of Schizophrenia Spectrum and Other Psychotic Disorders or Bipolar and Related Disorders, they were asked to select the specific diagnosis or diagnoses. Because the DSM-5-TR lists Schizotypal Personality Disorder under both the Schizophrenia Spectrum and Other Psychotic Disorders and Personality Disorders, participants were also asked to select the specific diagnosis or diagnoses if they reported a personal history of Personality Disorders. The same procedure was repeated at baseline with regard to the psychiatric history of family members (i.e. first-degree relatives and second-degree relatives). Because it is important to understand the relative contribution of varying degrees of genetic predisposition, we coded each psychiatric history variable (e.g. schizophrenia, bipolar I disorder) into four levels (0 = no psychiatric history, 1 = second-degree relative only, 2 = first-degree relative but no personal history, and 3 = personal history).

#### Psychotic symptoms

All participants were asked at both baseline and follow-up to complete a modified version of the Community Assessment of Psychic Experiences – Positive 15 (CAPE-P15) scale, which measures psychosis-like experiences (e.g. “have you heard voices when you are alone?”) that can be divided into three subscales: *persecutory ideation*, *perceptual abnormalities*, and *bizarre experiences.* The original CAPE-P15 scale asks participants about lifetime psychosis-like experiences (Capra, Kavanagh, Hides, & Scott, 2013), whereas other versions such as the Current CAPE-15 ask participants about recent psychosis-like experiences (e.g. in the past three months; Capra, Kavanagh, Hides, & Scott, 2017). In this study, we asked participants about psychosis-like experiences in the past week in order to increase the likelihood that the psychedelic use would precede the psychotic symptoms. Responses were rated on a Likert scale from 1 (“Never”) to 4 (“Nearly Always”). Internal consistency was excellent in the current sample (Cronbach’s alpha = 0.91 at baseline and 0.92 at non-imputed follow-up).

#### Manic symptoms

All participants were asked at both baseline and follow-up to complete the Altman Self-Rating Mania Scale (Altman, Hedeker, Peterson, & Davis, 1997), which assesses the presence and severity of manic (or hypomanic) symptoms using five items. The original questionnaire asks participants, for each item, to choose one of five statements (rated on a scale from 1 to 5) that best describes the way they have been feeling for the past week (e.g. “I feel happier or more cheerful than usual all of the time”), which was the same time frame used in this study. Internal consistency was acceptable in the current sample (Cronbach’s alpha = 0.78 at baseline and 0.80 at non-imputed follow-up).

### Statistical analyses

As specified in the preregistration, we used multiple linear regression to examine whether there were significant differences in changes (i.e. follow-up scores minus baseline scores) in the severity of psychotic or manic symptoms between participants who reported psychedelic use during the study period and those who did not. For each model, we conducted sensitivity analyses using follow-up scores as the outcome variable while controlling for baseline scores. This allowed us to assessed whether the observed results remained consistent when accounting for baseline levels.

We also used multiple linear regression to test whether there were interactions between psychedelic use during the study period and specific psychiatric histories (i.e. schizophrenia, bipolar I disorder) on changes in the severity of psychotic or manic symptoms. We were primarily interested in schizophrenia and bipolar I disorder, but exploratory analyses were conducted on other specific diagnoses under the Schizophrenia Spectrum and Other Psychotic Disorders, Bipolar and Related Disorders, and Personality Disorders. Additional exploratory analyses investigated associations between the legal status and frequency of psychedelic use and changes in the severity of psychotic or manic symptoms, as well as associations of challenging experiences and subjective experience of insight during the most intense psychedelic experience during the study period with changes in the severity of psychotic or manic symptoms.

All analyses included broadly the same covariates used in a recent longitudinal study that investigated psychedelic use and psychotic symptoms (Honk et al., 2024): age (recoded: 18–24, 25–34, 35–44, 45–50), gender identity (recoded: male, female, and other), educational attainment (bachelor’s degree or higher and less than bachelor’s degree), degree of religiosity (not at all religious, a little religious, quite religious, moderately religious, very religious), political affiliation (Democratic Party and Republican Party), past two-month use (yes, no) of alcohol, nicotine products, cannabis products, MDMA, major stimulants, illicit narcotic analgesics/opioids, illicit benzodiazepines and barbiturates, inhalants, and other substances at follow-up, as well as psychedelic use in the past two months at baseline. The analyses of challenging psychedelic experiences and psychedelic-related insights also controlled for dose used (low, moderate, large, very large, and extremely large) during participants’ most intense psychedelic experience during the study period. Because we collected data across two years (i.e. 2023 and 2024), we controlled for the survey year in all analyses.

To handle missing data at follow-up, we employed Multivariate Imputation by Chained Equations (MICE; van Buuren & Groothuis-Oudshoorn, 2011; mice package version 3.15.0 in R Studio) to impute the missing data across twenty imputed data sets using random forest imputations. Before imputing the data, we split the dataset into two subsets: (1) a subset with survey items only asked to participants who reported psychedelic use during the study period, which did not have any missing data; and (2) a subset with survey items asked to all participants (i.e. without any missing-by-design structures). Results were pooled using Rubin’s (1976) rules (i.e. across imputations and pooled using the “pool” function in the mice package; van Buuren & Groothuis-Oudshoorn, 2011). All continuous variables were standardized using the “scale” function. Descriptive statistics show non-imputed values only while results based on imputed data are presented for the multiple linear regression models unless otherwise specified. In all tests, a two-sided *p*<0.05 was used as the significance threshold. Because the study did not have predefined hypotheses and given the early, exploratory nature of work in this area, corrections for multiple comparisons were not applied to allow for the detection of potentially meaningful associations that could inform future research.

## Results

21,990 participants completed the survey at baseline and 12,345 of these participants completed the follow-up survey (56% retention). Among the participants who completed the follow-up survey, 505 reported psychedelic use during the two-month study period, which represents approximately 4% of the sample that completed the follow-up survey. Table 1 shows sample characteristics at baseline among participants who completed the follow-up survey. As shown in the table, compared with those who did not report psychedelic use during the two-month study, participants who reported psychedelic use during the study period had a higher lifetime history of psychedelic use (82% versus 21%) as well as other types of drug use (i.e. alcohol, nicotine products, cannabis products, MDMA, major stimulants, illicit narcotic analgesics or opioids, illicit benzodiazepines and barbiturates, inhalants, and other substances). There were also differences between groups regarding age, gender identity, degree of religiosity, and political affiliation. Notably, however, among those who reported psychedelic use during the study period, there were more individuals with a current or past history of bipolar I disorder. Finally, among those who reported psychedelic use during the study, their most intense psychedelic experience commonly involved psilocybin (69%), moderate or higher doses (66%), negative mindset prior to the experience (19%) or no psychological support during the experience (18%; see Table S1), and taking place in an illegal context (97% of full subsample; 100% and 95% of those with a personal history of schizophrenia and bipolar I disorder, respectively; see Table S2).

**Table 1.** Sample characteristics

	Psychedelic use during the study period		
	Yes (*n* =505)	No (*n* = 11,840)	*p*
**Age**			.024
18–24	21.0%	17.5%	
25–34	41.4%	40.1%	
35–44	28.9%	30.0%	
45–50	8.7%	12.5%	
**Gender identity**			<.001
Male	45.7%	35.6%	
Female	49.1%	59.8%	
Other	5.2%	4.6%	
**Education**			.067
Bachelor’s degree or higher	52.5%	56.6%	
Less than bachelor’s degree	47.5%	43.4%	
**Degree of religiosity**			.010
Not at all religious	53.2%	46.7%	
A little religious	21.8%	22.4%	
Quite religious	13.5%	15.3%	
Moderately religious	6.9%	10.0%	
Very religious	4.6%	6.6%	
**Political affiliation**			.007
Democratic party	79.2%	73.8%	
Republican party	20.8%	26.2%	
**Lifetime substance use**			
Psychedelics	82.0%	21.1%	<.001
Alcohol	88.1%	77.5%	<.001
Nicotine products	74.3%	47.9%	<.001
Cannabis products	87.9%	63.0%	<.001
MDMA	48.1%	14.2%	<.001
Major stimulants	50.9%	16.7%	<.001
Illicit narcotic analgesics or opioids	29.1%	11.0%	<.001
Illicit benzodiazepines and barbiturates	37.6%	14.0%	<.001
Inhalants	32.7%	8.7%	<.001
Other substances	13.3%	3.9%	<.001
**Personal psychiatric history**			
Schizophrenia	0.8%	0.4%	.162
Bipolar I disorder	4.4%	1.7%	<.001

This table shows sample characteristics at baseline of participants who completed the follow-up survey and reported psychedelic use during the study period and participants who completed the follow-up survey and did not report psychedelic use during the same period. All percentages were calculated based on the total number (n) for each column and were rounded to the nearest 0.1%; cumulative percentages may not add to 100.0. Pearson’s chi-squared tests were used to examine the characteristics of psychedelic users versus non-users.

Table 2 shows results from the multiple linear regression models on the associations between psychedelic use during the study period and changes in the severity of psychotic and manic symptoms, including the models that evaluate interactions of psychedelic use with a psychiatric history of schizophrenia or bipolar I disorder. As demonstrated in the table, psychedelic use during the study period was associated with increases in the severity of both psychotic and manic symptoms (*β*=0.35, *p*<.001; *β*=0.14, *p*=.006; see Table 2 and Tables S3 and S4). However, compared with participants who did not report psychedelic use during the study period, exploratory analyses revealed that increases in the severity of both psychotic and manic symptoms were only observed among participants who reported psychedelic use in an illegal context (*β*=0.37, *p*<.001; *β*=0.15, *p*=.004; see Table S5). Exploratory analyses also suggested that the association between psychedelic use and psychotic (but not manic) symptoms was frequency-dependent (i.e. more frequent use associated with greater increases), at least for certain aspects of psychotic symptoms (e.g. perceptual abnormalities; see Table S6). Further exploratory analyses showed that more severe challenging psychedelic experiences were associated with increases in the severity of psychotic (but not manic) symptoms among those who reported psychedelic use during the study period (*β*=0.34, *p*<.001). At the same time, greater psychedelic-related insights were associated with increases in the severity of manic (but not psychotic) symptoms among those who reported psychedelic use during the study period (*β*=0.14, *p*=.015; see Table S7).

**Table 2.** Model estimates

Change in severity of psychotic symptoms			
Predictor variable	Interaction term	*β* (SE)	*p*
Psychedelic use	…	0.35 (0.05)	<.001
Psychedelic use	No SZC history	…	…
	2nd-degree SCZ history	−0.06 (0.19)	.749
	1st-degree SCZ history	0.04 (0.24)	.856
	Personal SCZ history	−0.75 (0.52)	.153
Psychedelic use	No BIP history	…	…
	2nd-degree BIP history	0.32 (0.28)	.260
	1st-degree BIP history	0.10 (0.16)	.549
	Personal BIP history	0.08 (0.23)	.715

Change in severity of manic symptoms			
Predictor variable	Interaction term	*β* (SE)	*p*
Psychedelic use	…	0.14 (0.05)	.006
Psychedelic use	No SZC history	…	…
	2nd-degree SCZ history	0.05 (0.19)	.789
	1st-degree SCZ history	−0.09 (0.24)	.719
	Personal SCZ history	1.14 (0.52)	.027
Psychedelic use	No BIP history	…	…
	2nd-degree BIP history	0.48 (0.29)	.095
	1st-degree BIP history	−0.10 (0.16)	.528
	Personal BIP history	0.68 (0.23)	.003

This table shows associations between psychedelic use and changes in severity of psychotic or manic symptoms, as well as interactions between psychedelic use and specific psychiatric histories (i.e. schizophrenia, bipolar I disorder) on changes in severity of psychotic or manic symptoms. β = standardized coefficient; SE = standard error; p = p-value; SCZ = schizophrenia; BIP = bipolar I disorder. The models controlled for age, gender identity, educational attainment, degree of religiosity, political affiliation, past two-month use of alcohol, nicotine products, cannabis products, MDMA, major stimulants, illicit narcotic analgesics/opioids, illicit benzodiazepines and barbiturates, inhalants, and other substances at follow-up, psychedelic use in the past two months at baseline, and survey year. See Table S9 for results from the non-imputed data.

In the interaction models, a personal history of schizophrenia or bipolar I disorder interacted with psychedelic use on manic (but not psychotic) symptoms such that those with a personal history of these disorders who used psychedelics reported greater increases in the severity of manic symptoms (*β*=1.14, *p*=.027; *β*=0.68, *p*=.003; see Table 2 and Tables S3 and S4). Notably, although there were no significant interactions, for example, with conditions such as bipolar II disorder, additional exploratory analyses revealed that individuals with certain other psychiatric histories (e.g. schizotypal personality disorder; *β*=1.57, *p*=.015) who used psychedelics reported greater increases in the severity of manic symptoms (see Table S8). The results were broadly consistent in sensitivity analyses.

## Discussion

This study investigated the longitudinal associations of naturalistic psychedelic use with psychotic and manic symptoms, especially among putatively vulnerable individuals with a psychiatric history of schizophrenia or bipolar I disorder. The study findings showed associations between naturalistic psychedelic use and increases in the severity of both psychotic and manic symptoms, with such increases only observed for those who reported naturalistic psychedelic use in an illegal context. While increases in the severity of psychotic symptoms appeared to depend on more frequent use and more severe challenging psychedelic experiences, increases in the severity of manic symptoms appeared to be moderated by a personal history of schizophrenia or bipolar I disorder and greater subjective experience of insight during a psychedelic experience. Notably, individuals with certain other psychiatric histories (e.g. schizotypal personality disorder) who used psychedelics also reported greater increases in the severity of manic symptoms.

The pattern of only observing increases in the severity of both psychotic and manic symptoms among those who reported naturalistic psychedelic use in an illegal context could have several potential explanations. For example, individuals who use psychedelic in an illegal context may generally experience more stress or anxiety during the psychedelic experience due to fear of legal repercussions, which may negatively impact post-acute outcomes. It is also possible that differences in the purity and potency of psychedelic substances used in illegal versus non-illegal contexts could partially explain the results. Future research should further interrogate this.

The association between naturalistic psychedelic use and increases in the severity of psychotic symptoms in this study contrasts with a recent longitudinal study that reported decreases in delusional ideation following naturalistic psychedelic use (Zhou, De Wied, Carhart-Harris, & Kettner, 2022; see also Honk et al., 2024; Simonsson, Mosing, et al., 2024b). These differences may be explained by differences across studies in sample characteristics, research design, or measurement of psychotic symptoms. It is possible, for instance, that the samples differed in knowledge about psychedelic harm reduction or exposure to extra-pharmacological factors that have been associated with challenging psychedelic experiences in previous research (e.g. lack of psychological support; Simonsson, Hendricks, et al., 2023c). Such an explanation seems reasonable in light of the association in this study between the severity of challenging psychedelic experiences and increases in the severity of psychotic symptoms. In fact, a large portion of participants in the current study who reported psychedelic use also reported that many such extra-pharmacological factors were present during their most intense psychedelic experience during the study period, which indicates that circumstances may not have been ideal for positive outcomes in this sample. There did appear to be a frequency-dependent relationship between psychedelic use and psychotic symptoms, though, such that the more times psychedelics had been used in the study period, the larger the effect sizes. If there is indeed a frequency-dependent relationship between psychedelic use and psychotic symptoms, such findings could potentially be explained by a greater likelihood of having a more severe challenging psychedelic experience among those who used psychedelics on more occasions. Future research should explore this further.

Notably, however, individuals with a personal history of schizophrenia or bipolar I disorder did not appear to be at risk for increases in the severity of psychotic symptoms as a result of psychedelic use. Such null findings correspond with results from a recent cross-sectional study that evaluated interactions between polygenic scores for schizophrenia or bipolar I disorder and lifetime psychedelic use on self-reported psychotic symptoms among adolescents (Simonsson, Mosing, et al., 2024b; see also Sapienza et al., 2025). At the same time, the present findings contrast with results from a recent longitudinal study that evaluated interactions with a personal history of psychotic or bipolar disorders more generally and naturalistic psychedelic use during the study period on psychotic symptoms (Honk et al., 2024). It is possible that the contrast in findings can be explained by differences across studies in the measurement of psychotic symptoms (e.g. Current CAPE-15 versus psychotic ideation subscale of the Psychiatric Diagnostic Screening Questionnaire; Zimmerman & Mattia, 2001) or the specificity of disorder categorization (e.g. schizophrenia specifically versus psychotic disorders more generally, bipolar I disorder specifically versus bipolar disorders more generally). Future research on psychedelic use and psychotic symptoms should aim for more consistency with regard to assessment procedures and more specificity with regard to disorder categorization.

Although the finding that naturalistic psychedelic use and the subjective experience of insight during a psychedelic experience were longitudinally associated with greater increases in the severity of manic symptoms is relatively novel, the results on manic symptoms from interaction models in this study partially replicate previous findings (Simonsson, Mosing, et al., 2024b). These findings suggest that a personal history of schizophrenia or bipolar I disorder may indeed be contraindicated for psychedelic use (Johnson et al., 2008). Notably, however, those with a personal history of schizotypal personality disorder who used psychedelics also reported greater increases in the severity of manic symptoms. This supports the possibility that a specific genetic profile, more prevalent in certain psychiatric populations, may be contraindicated for psychedelic use. It is important to note, however, that among participants who reported psychedelic use in a non-illegal context during the study period, barely anyone had a personal history of schizophrenia (*n*=0), bipolar I disorder (*n*=1), or schizotypal personality disorder (*n*=0), which could potentially explain the greater increase in manic symptoms in these populations. Future research should nonetheless further explore the possibility of developing genetic biomarkers that can be used to recommend for or against psychedelic use.

### Limitations

This study has several limitations that should be considered. First, the study used purposive sampling to maximize the number of participants who were likely to report psychedelic use during the study period. This may limit generalizations to the population at large. Second, there was only a small number of participants who reported both psychedelic use during the study period and a personal history of schizophrenia (*n*=4) or bipolar I disorder (*n*=22). Similarly, only a small number of participants reported psychedelic use in a non-illegal context (*n*=33). Results drawn from such a small proportion of the study sample should be interpreted cautiously. Third, the attrition rate at follow-up was relatively high. Fourth, no corrections were made for multiple comparisons, which increases the risk of Type 1 errors, though the main analyses were pre-registered. Fifth, due to the study design (i.e. observational), no conclusive causal inferences can be made. Sixth, given that all responses were self-reported, it is possible that recall or social desirability biases, for example, led participants to inaccurately report the types of substances used or psychiatric history and symptoms. Seventh, there was also no assessment of the number of relatives with a possible psychiatric history, which could have been important for understanding genetic vulnerability. Eighth, this study did not evaluate the clinical meaning of the significant associations. It is possible, for instance, that increases in psychotic symptoms reflect a beneficial change in creativity or that increases in manic symptoms capture improvements in mood from a previously depressed state (Dourron, Strauss, & Hendricks, 2022). This should be investigated in future research. Future longitudinal studies should also investigate interactions between psychedelic use and self-reported psychiatric history or polygenic scores for schizophrenia, bipolar I disorder, or other disorders of concern (e.g. schizotypal personality disorder) on psychotic or manic symptoms, ideally with symptoms rated by a clinician. This work is crucial for ensuring that the risks associated with psychedelic use, both in naturalistic and clinical settings, can be minimized and mitigated.

## Supporting information

Simonsson et al. supplementary material Simonsson et al. supplementary material

## Data Availability

The unimputed data and R syntax for primary analyses are available at https://doi.org/10.6084/m9.figshare.28645415.v1.

## Supplementary material

The supplementary material for this article can be found at http://doi.org/10.1017/S0033291725000716.
