# Supplementary material for: Longitudinal associations of naturalistic psychedelic use with psychotic and manic symptoms
Source: Psychol Med. 2025 Mar 31;55:e99. doi: 10.1017/S0033291725000716 (PMC12094617; doi:10.1017/S0033291725000716)
Supplement: Simonsson et al. supplementary material [file S0033291725000716sup001.docx]

***Supplemental Materials***

**List of Survey Items on Demographics**

What is your age?

18-50

What is your gender?

- Male
- Female
- Transgender (male to female)
- Transgender (female to male)
- Non-binary gender
- Other

Do you have a bachelor's degree or higher?

- Yes
- No

How religious are you?

- Not at all religious
- A little religious
- Moderately religious
- Quite religious
- Very religious

If you had to choose, do you think of yourself as closer to the Democratic Party or the Republican Party?

- Democratic Party
- Republican Party

**List of DSM-5-TR Items**

Do you have… Do you have a first-degree relative (e.g., parents, siblings) with… Do you have a second-degree relative (e.g., grandparents, uncles or aunts) with… a current or past diagnosis of any of the following mental disorders (select all that apply)?

- Schizophrenia Spectrum and Other Psychotic Disorders
- Bipolar and Related Disorders
- Depressive Disorders
- Anxiety Disorders
- Obsessive-Compulsive and Related Disorders
- Trauma- and Stressor-Related Disorders
- Dissociative Disorders
- Somatic Symptom and Related Disorders
- Feeding and Eating Disorders
- Sleep-Wake Disorders
- Substance-Related and Addictive Disorders
- Personality Disorders
- Neurodevelopmental Disorders
- Elimination Disorders
- Sexual Dysfunctions
- Gender Dysphoria
- Disruptive, Impulse-Control, and Conduct Disorders
- Neurocognitive Disorders
- Paraphilic Disorders
- Other Mental Disorders
- None of the above

You reported having… a first-degree relative (e.g., parents, siblings) with… a second-degree relative (e.g., grandparents, uncles or aunts) with… a current or past diagnosis of schizophrenia spectrum and other psychotic disorders. Please specify which of the following that [you, they] have a current or past diagnosis of (select all that apply).

- Schizotypal Personality Disorder
- Delusional Disorder
- Brief Psychotic Disorder
- Schizophreniform Disorder
- Schizophrenia
- Schizoaffective Disorder
- Substance/Medication-Induced Psychotic Disorder
- Psychotic Disorder Due to Another Medical Condition
- Catatonia Associated With Another Mental Disorder (Catatonia Specifier)
- Catatonic Disorder Due to Another Medical Condition
- Unspecified Catatonia
- Other Specified Schizophrenia Spectrum and Other Psychotic Disorder
- Unspecified Schizophrenia Spectrum and Other Psychotic Disorder
- None of the above

You reported having… a first-degree relative (e.g., parents, siblings) with… a second-degree relative (e.g., grandparents, uncles or aunts) with… a current or past diagnosis of bipolar and related disorders. Please specify which of the following that [you, they] have a current or past diagnosis of (select all that apply).

- Bipolar I Disorder
- Bipolar II Disorder
- Cyclothymic Disorder
- Substance/Medication-Induced Bipolar and Related Disorder
- Bipolar and Related Disorder Due to Another Medical Condition
- Other Specified Bipolar and Related Disorder
- Unspecified Bipolar and Related Disorder
- None of the above

You reported having… a first-degree relative (e.g., parents, siblings) with… a second-degree relative (e.g., grandparents, uncles or aunts) with… a current or past diagnosis of personality disorders. Please specify which of the following that [you, they] have a current or past diagnosis of (select all that apply).

- Paranoid Personality Disorder
- Schizoid Personality Disorder
- Schizotypal Personality Disorder
- Antisocial Personality Disorder
- Borderline Personality Disorder
- Histrionic Personality Disorder
- Narcissistic Personality Disorder
- Avoidant Personality Disorder
- Dependent Personality Disorder
- Obsessive-Compulsive Personality Disorder
- Personality Change Due to Another Medical Condition
- Other Specified Personality Disorder
- Unspecified Personality Disorder
- None of the above

| **Table S1.** | **%** |  |  |  |
| --- | --- | --- | --- | --- |
|  |  | ***Psychedelic use in illegal context*** | | |
|  | Total (n=505) | No (n=33) | Yes (n=472) | *p* |
| ***Type of psychedelic used*** |  |  |  |  |
| Ayahuasca | 2% | 3% | 2% | .729 |
| DMT | 10% | 9% | 10% | .933 |
| Psilocybin | 69% | 91% | 67% | .004 |
| LSD | 20% | 0% | 22% | .003 |
| Mescaline | 7% | 0% | 7% | .105 |
| Peyote | 2% | 0% | 2% | .375 |
| San Pedro | 1% | 0% | 1% | .646 |
| ***Type of dose used*** | |  |  | .794 |
| Low | 34% | 39% | 34% |  |
| Moderate | 47% | 39% | 47% |  |
| Large | 12% | 15% | 11% |  |
| Very large | 5% | 6% | 5% |  |
| Extremely large | 2% | 0% | 2% |  |
| ***Extra-pharmacological factors*** | |  |  |  |
| Insufficient or inadequate preparation for the experience (e.g., took substance without knowing what to expect) | 10% | 9% | 10% | .872 |
| Major life event prior to the experience (e.g., death of a loved one, divorce) | 11% | 12% | 10% | .753 |
| Negative mindset prior to the experience (e.g., fear, anxiety, anger) | 19% | 21% | 19% | .715 |
| No psychological support present during the experience | 18% | 6% | 18% | .071 |
| Disagreeable or uncomfortable physical environment | 12% | 12% | 13% | .949 |
| Disagreeable or uncomfortable musical environment | 5% | 6% | 5% | .850 |
| Disagreeable or uncomfortable social environment | 9% | 12% | 8% | .443 |
| Dose was too large | 5% | 3% | 5% | .664 |
| Combining a classic psychedelic with another drug | 7% | 9% | 7% | .576 |
| Other | 4% | 9% | 3% | .077 |
| This table shows variables associated with most intense psychedelic experience during the study period among those who reported psychedelic use (n=505). All percentages were calculated based on the total number (n) for each column and were rounded to the nearest 1%; cumulative percentages may not add to 100.0. The items with psychedelic substance type, as well as extra-pharmacological factors, were multiple-choice and participants could endorse any and all responses that applied. Pearson's chi-squared tests were used to examine the characteristics of those who reported psychedelic use in illegal context versus those who reported psychedelic use in non-illegal context. | | | | |

| **Table S2** | | | |
| --- | --- | --- | --- |
|  | Personal history of SCZ (n=4) | Personal history of BIP (n=22) | All (n=505) |
| Psychedelic use in illegal context | 100% | 95% | 93% |
| Psychedelic use in non-illegal context | 0% | 5% | 7% |
| This table shows legal status of psychedelic use associated with most intense psychedelic experience during the study period among those who reported psychedelic use. All percentages were calculated based on the total number (n) for each column and were rounded to the nearest 1%. SCZ = schizophrenia, BIP = bipolar I disorder. | | | |

| **Table S3.** | | | |
| --- | --- | --- | --- |
| Change in severity of persecutory ideation | | | |
| Predictor variable | Interaction term | β (SE) | p |
| Psychedelic use | … | 0.23 (0.05) | <.001 |
| Psychedelic use | No SZC history | … | … |
|  | 2^nd^ degree SCZ history | 0.08 (0.19) | .688 |
|  | 1^st^ degree SCZ history | 0.07 (0.24) | .769 |
|  | Personal SCZ history | -0.79 (0.52) | .126 |
| Psychedelic use | No BIP history | … | … |
|  | 2^nd^ degree BIP history | 0.19 (0.28) | .503 |
|  | 1^st^ degree BIP history | -0.13 (0.16) | .423 |
|  | Personal BIP history | 0.18 (0.23) | .440 |
| Change in severity of perceptual abnormalities | | | |
| Predictor variable | Interaction term | β (SE) | p |
| Psychedelic use | … | 0.38 (0.05) | <.001 |
| Psychedelic use | No SZC history | … | … |
|  | 2^nd^ degree SCZ history | 0.11 (0.19) | .549 |
|  | 1^st^ degree SCZ history | 0.28 (0.24) | .248 |
|  | Personal SCZ history | -1.35 (0.53) | .010 |
| Psychedelic use | No BIP history | … | … |
|  | 2^nd^ degree BIP history | 0.10 (0.28) | .725 |
|  | 1^st^ degree BIP history | 0.27 (0.16) | .098 |
|  | Personal BIP history | 0.32 (0.23) | .155 |
| Change in severity of bizarre experiences | | | |
| Predictor variable | Interaction term | β (SE) | p |
| Psychedelic use | … | 0.28 (0.05) | <.001 |
| Psychedelic use | No SZC history | … | … |
|  | 2^nd^ degree SCZ history | -0.21 (0.19) | .258 |
|  | 1^st^ degree SCZ history | -0.11 (0.24) | .634 |
|  | Personal SCZ history | -0.08 (0.53) | .884 |
| Psychedelic use | No BIP history | … | … |
|  | 2^nd^ degree BIP history | 0.39 (0.28) | .170 |
|  | 1^st^ degree BIP history | 0.20 (0.16) | .203 |
|  | Personal BIP history | -0.16 (0.23) | .480 |
| This table shows interactions between psychedelic use and specific psychiatric histories (i.e., schizophrenia, bipolar I disorder) on changes in severity of persecutory ideation, perceptual abnormalities, and bizarre experiences (i.e., subscales of psychotic symptoms). β = standardized coefficient; SE = standard error; p = p-value; SCZ = schizophrenia, BIP = bipolar I disorder. The models controlled for age, gender identity, educational attainment, degree of religiosity, political affiliation, past two-month use of alcohol, nicotine products, cannabis products, MDMA, major stimulants, illicit narcotic analgesics/opioids, illicit benzodiazepines and barbiturates, inhalants, and other substances at follow-up, psychedelic use in the past two months at baseline, and survey year. | | | |

| **Table S4.** | **n** | **Baseline** | **Follow-up** | **n** | **Baseline** | **Follow-up** |
| --- | --- | --- | --- | --- | --- | --- |
|  | Personal history of schizophrenia (Yes) | | | Personal history of schizophrenia (No) | | |
|  |  | Psychotic symptoms | |  | Psychotic symptoms | |
| Psychedelic use | 4 | 35.50 (8.06) | 29.00 (9.42) | 501 | 22.85 (8.32) | 23.39 (9.44) |
| No psychedelics use | 46 | 33.46 (12.32) | 31.07 (11.52) | 11,794 | 19.51 (5.94) | 19.33 (6.03) |
|  |  | Persecutory ideation | |  | Persecutory ideation | |
| Psychedelic use | 4 | 14.00 (1.83) | 11.25 (4.19) | 501 | 8.80 (3.26) | 8.95 (3.51) |
| No psychedelics use | 46 | 12.15 (4.51) | 11.04 (4.15) | 11,794 | 7.60 (2.81) | 7.53 (2.85) |
|  |  | Perceptual abnormalities | |  | Perceptual abnormalities | |
| Psychedelic use | 4 | 7.50 (1.29) | 5.50 (2.38) | 501 | 3.94 (1.84) | 4.21 (2.15) |
| No psychedelics use | 46 | 7.09 (3.32) | 6.96 (3.24) | 11,794 | 3.38 (1.16) | 3.38 (1.18) |
|  |  | Bizarre experiences | |  | Bizarre experiences | |
| Psychedelic use | 4 | 14.00 (6.48) | 12.25 (4.11) | 501 | 10.11 (4.26) | 10.24 (4.75) |
| No psychedelics use | 46 | 14.22 (5.73) | 13.07 (5.69) | 11,794 | 8.53 (2.94) | 8.42 (2.91) |
|  |  | Manic symptoms | |  | Manic symptoms | |
| Psychedelic use | 4 | 9.75 (2.06) | 13.25 (6.80) | 501 | 9.86 (3.91) | 10.01 (3.95) |
| No psychedelics use | 46 | 10.63 (3.99) | 9.57 (3.77) | 11,794 | 9.27 (3.74) | 9.11 (3.73) |
|  | Personal history of bipolar I disorder (Yes) | | | Personal history of bipolar I disorder (No) | | |
|  |  | Psychotic symptoms | |  | Psychotic symptoms | |
| Psychedelic use | 22 | 29.05 (11.27) | 28.82 (11.71) | 483 | 22.67 (8.14) | 23.19 (9.27) |
| No psychedelics use | 200 | 23.30 (8.28) | 22.66 (7.90) | 11,640 | 19.50 (5.97) | 19.32 (6.06) |
|  |  | Persecutory ideation | |  | Persecutory ideation | |
| Psychedelic use | 22 | 10.68 (3.92) | 10.95 (4.38) | 483 | 8.76 (3.24) | 8.88 (3.45) |
| No psychedelics use | 200 | 9.52 (3.72) | 9.13 (3.62) | 11,640 | 7.59 (2.81) | 7.52 (2.85) |
|  |  | Perceptual abnormalities | |  | Perceptual abnormalities | |
| Psychedelic use | 22 | 5.18 (2.32) | 5.50 (2.96) | 483 | 3.91 (1.82) | 4.16 (2.10) |
| No psychedelics use | 200 | 4.02 (1.81) | 4.02 (1.94) | 11,640 | 3.38 (1.19) | 3.38 (1.20) |
|  |  | Bizarre experiences | |  | Bizarre experiences | |
| Psychedelic use | 22 | 13.18 (5.94) | 12.36 (5.96) | 483 | 10.00 (4.15) | 10.16 (4.66) |
| No psychedelics use | 200 | 9.77 (4.02) | 9.52 (3.75) | 11,640 | 8.53 (2.95) | 8.42 (2.92) |
|  |  | Manic symptoms | |  | Manic symptoms | |
| Psychedelic use | 22 | 9.50 (4.31) | 11.95 (4.46) | 483 | 9.87 (3.88) | 9.95 (3.94) |
| No psychedelics use | 200 | 9.26 (3.65) | 8.88 (3.47) | 11,640 | 9.28 (3.75) | 9.12 (3.74) |
| This table shows unadjusted descriptive statistics on outcome variables among participants who completed the follow-up survey. Number of participants (n) in each category. Mean scores (standard deviation) at baseline and follow-up. | | | | | | |

| **Table S5.** | | |
| --- | --- | --- |
| Change in severity of psychotic symptoms | | |
|  | β (SE) | p |
| No psychedelic use | … | … |
| Used in illegal context | 0.37 (0.05) | <.001 |
| Used in non-illegal context | 0.10 (0.18) | .577 |
| Used in illegal context | … | … |
| No psychedelic use | -0.37 (0.05) | <.001 |
| Used in non-illegal context | -0.27 (0.18) | .135 |
| Change in severity of persecutory ideation | | |
|  | β (SE) | p |
| No psychedelic use | … | … |
| Used in illegal context | 0.24 (0.05) | <.001 |
| Used in non-illegal context | 0.11 (0.17) | .536 |
| Used in illegal context | … | … |
| No psychedelic use | -0.24 (0.05) | <.001 |
| Used in non-illegal context | -0.13 (0.18) | .457 |
| Change in severity of perceptual abnormalities | | |
|  | β (SE) | p |
| No psychedelic use | … | … |
| Used in illegal context | 0.41 (0.05) | <.001 |
| Used in non-illegal context | 0.00 (0.18) | .997 |
| Used in illegal context | … | … |
| No psychedelic use | -0.41 (0.05) | <.001 |
| Used in non-illegal context | -0.41 (0.18) | .024 |
| Change in severity of bizarre experiences | | |
|  | β (SE) | p |
| No psychedelic use | … | … |
| Used in illegal context | 0.30 (0.05) | <.001 |
| Used in non-illegal context | 0.09 (0.18) | .604 |
| Used in illegal context | … | … |
| No psychedelic use | -0.30 (0.05) | <.001 |
| Used in non-illegal context | -0.21 (0.18) | .251 |
| Change in severity of manic symptoms | | |
|  | β (SE) | p |
| No psychedelic use | … | … |
| Used in illegal context | 0.15 (0.05) | .004 |
| Used in non-illegal context | -0.03 (0.18) | .880 |
| Used in illegal context | … | … |
| No psychedelic use | -0.15 (0.05) | .004 |
| Used in non-illegal context | -0.18 (0.18) | .315 |
| This table shows associations between legal status of psychedelic use and changes in the severity of psychotic or manic symptoms. The reference category for the categorical predictor has been altered across models with each reference category highlighted with three dots in the β (SE) and p columns (…). β = standardized coefficient; SE = standard error; p = p-value. The linear regression models controlled for age, gender identity, educational attainment, degree of religiosity, political affiliation, past two-month use of alcohol, nicotine products, cannabis products, MDMA, major stimulants, illicit narcotic analgesics/opioids, illicit benzodiazepines and barbiturates, inhalants, and other substances at follow-up, psychedelic use in the past two months at baseline, and survey year. | | |

| **Table S6.** | | |
| --- | --- | --- |
| Change in severity of psychotic symptoms | | |
|  | β (SE) | p |
| No psychedelic use | … | … |
| Used once | 0.24 (0.07) | <.001 |
| Used twice | 0.38 (0.09) | <.001 |
| Used 3 times or more | 0.61 (0.11) | <.001 |
| Used once | … | … |
| No psychedelic use | -0.24 (0.07) | <.001 |
| Used twice | 0.14 (0.10) | .190 |
| Used 3 times or more | 0.37 (0.12) | .002 |
| Used twice | … | … |
| No psychedelic use | -0.38 (0.09) | <.001 |
| Used once | -0.14 (0.10) | .190 |
| Used 3 times or more | 0.23 (0.13) | .077 |
| Change in severity of persecutory ideation | | |
|  | β (SE) | p |
| No psychedelic use | … | … |
| Used once | 0.17 (0.07) | .012 |
| Used twice | 0.25 (0.09) | .005 |
| Used 3 times or more | 0.40 (0.11) | <.001 |
| Used once | … | … |
| No psychedelic use | -0.17 (0.07) | .012 |
| Used twice | 0.08 (0.10) | .423 |
| Used 3 times or more | 0.23 (0.12) | .053 |
| Used twice | … | … |
| No psychedelic use | -0.25 (0.09) | .005 |
| Used once | -0.08 (0.10) | .423 |
| Used 3 times or more | 0.15 (0.13) | .254 |
| Change in severity of perceptual abnormalities | | |
|  | β (SE) | p |
| No psychedelic use | … | … |
| Used once | 0.24 (0.07) | <.001 |
| Used twice | 0.45 (0.09) | <.001 |
| Used 3 times or more | 0.68 (0.11) | <.001 |
| Used once | … | … |
| No psychedelic use | -0.24 (0.07) | <.001 |
| Used twice | 0.21 (0.10) | .004 |
| Used 3 times or more | 0.44 (0.12) | <.001 |
| Used twice | … | … |
| No psychedelic use | -0.45 (0.09) | <.001 |
| Used once | -0.21 (0.10) | .040 |
| Used 3 times or more | 0.23 (0.13) | .080 |
| Change in severity of bizarre experiences | | |
|  | β (SE) | p |
| No psychedelic use | … | … |
| Used once | 0.21 (0.07) | .002 |
| Used twice | 0.30 (0.09) | <.001 |
| Used 3 times or more | 0.49 (0.11) | <.001 |
| Used once | … | … |
| No psychedelic use | -0.21 (0.07) | .002 |
| Used twice | 0.09 (0.10) | .393 |
| Used 3 times or more | 0.29 (0.12) | .018 |
| Used twice | … | … |
| No psychedelic use | -0.30 (0.09) | <.001 |
| Used once | -0.09 (0.10) | .393 |
| Used 3 times or more | 0.20 (0.13) | .130 |
| Change in severity of manic symptoms | | |
|  | β (SE) | p |
| No psychedelic use | … | … |
| Used once | 0.11 (0.07) | .117 |
| Used twice | 0.22 (0.09) | .012 |
| Used 3 times or more | 0.13 (0.11) | .233 |
| Used once | … | … |
| No psychedelic use | -0.11 (0.07) | .117 |
| Used twice | 0.11 (0.10) | .281 |
| Used 3 times or more | 0.02 (0.12) | .850 |
| Used twice | … | … |
| No psychedelic use | -0.22 (0.09) | .012 |
| Used once | -0.11 (0.10) | .281 |
| Used 3 times or more | -0.09 (0.13) | .495 |
| This table shows associations between frequency of psychedelic use and changes in the severity of psychotic or manic symptoms. The reference category for the categorical predictor has been altered across models with each reference category highlighted with three dots in the β (SE) and p columns (…). β = standardized coefficient; SE = standard error; p = p-value. The linear regression models controlled for age, gender identity, educational attainment, degree of religiosity, political affiliation, past two-month use of alcohol, nicotine products, cannabis products, MDMA, major stimulants, illicit narcotic analgesics/opioids, illicit benzodiazepines and barbiturates, inhalants, and other substances at follow-up, psychedelic use in the past two months at baseline, and survey year. | | |

| **Table S7.** | | |
| --- | --- | --- |
| Change in severity of psychotic symptoms | | |
|  | β (SE) | p |
| CEQ total score | 0.34 (0.07) | <.001 |
| PIQ total score | 0.01 (0.07) | .827 |
| Change in severity of persecutory ideation | | |
|  | β (SE) | p |
| CEQ total score | 0.28 (0.06) | <.001 |
| PIQ total score | -0.07 (0.06) | .246 |
| Change in severity of perceptual abnormalities | | |
|  | β (SE) | p |
| CEQ total score | 0.28 (0.08) | <.001 |
| PIQ total score | 0.11 (0.08) | .162 |
| Change in severity of bizarre experiences | | |
|  | β (SE) | p |
| CEQ total score | 0.27 (0.07) | <.001 |
| PIQ total score | 0.04 (0.07) | .497 |
| Change in severity of manic symptoms | | |
|  | β (SE) | p |
| CEQ total score | -0.05 (0.06) | .379 |
| PIQ total score | 0.14 (0.06) | .015 |
| This table shows associations between the acute psychedelic experience and changes in the severity of psychotic or manic symptoms. β = standardized coefficient; SE = standard error; p = p-value. The linear regression models controlled for age, gender identity, educational attainment, degree of religiosity, political affiliation, past two-month use of alcohol, nicotine products, cannabis products, MDMA, major stimulants, illicit narcotic analgesics/opioids, illicit benzodiazepines and barbiturates, inhalants, and other substances at follow-up, psychedelic use in the past two months at baseline, dose used during most intense psychedelic experience during the study period, and survey year. | | |

| **Table S8.** | | | |
| --- | --- | --- | --- |
| Change in severity of psychotic symptoms | | | |
| Predictor variable | Interaction term | β (SE) | p |
| Psychedelic use | No SMIBRD history | … | … |
|  | 2^nd^ degree SMIBRD history | 1.12 (0.39) | .004 |
|  | 1^st^ degree SMIBRD history | 0.40 (0.26) | .117 |
|  | Personal SMIBRD history | -0.45 (0.42) | .290 |
| Psychedelic use | No BIIP history | … | … |
|  | 2^nd^ degree BIIP history | -0.39 (0.28) | .163 |
|  | 1^st^ degree BIIP history | -0.11 (0.22) | .625 |
|  | Personal BIIP history | 0.14 (0.21) | .503 |
| Psychedelic use | No BRDDAMC history | … | … |
|  | 2^nd^ degree BRDDAMC history | 0.46 (0.44) | .300 |
|  | 1^st^ degree BRDDAMC history | 0.52 (0.52) | .315 |
|  | Personal BRDDAMC history | -0.32 (0.61) | .598 |
| Psychedelic use | No UBRD history | … | … |
|  | 2^nd^ degree UBRD history | -0.18 (0.23) | .437 |
|  | 1^st^ degree UBRD history | -0.34 (0.20) | .873 |
|  | Personal UBRD history | 0.42 (0.37) | .262 |
| Psychedelic use | No BPD history | … | … |
|  | 2^nd^ degree BPD history | -1.15 (0.42) | .006 |
|  | 1^st^ degree BPD history | -0.20 (0.25) | .429 |
|  | Personal BPD history | 0.27 (0.23) | .254 |
| Psychedelic use | No OCPD history | … | … |
|  | 2^nd^ degree OCPD history | -1.22 (0.39) | .002 |
|  | 1^st^ degree OCPD history | -0.12 (0.33) | .710 |
|  | Personal OCPD history | -0.17 (0.59) | .745 |
| Psychedelic use | No SCZPD history | … | … |
|  | 2^nd^ degree SCZPD history | -0.71 (0.42) | .091 |
|  | 1^st^ degree SCZPD history | 0.14 (0.43) | .748 |
|  | Personal SCZPD history | -0.18 (0.64) | .780 |
| Psychedelic use | No SCZAD history | … | … |
|  | 2^nd^ degree SCZAD history | 1.32 (0.59) | .026 |
|  | 1^st^ degree SCZAD history | 0.15 (0.39) | .695 |
|  | Personal SCZAD history | -0.16 (0.48) | .742 |
| Change in severity of persecutory ideation | | | |
| Predictor variable | Interaction term | β (SE) | p |
| Psychedelic use | No SMIBRD history | … | … |
|  | 2^nd^ degree SMIBRD history | 0.82 (0.39) | .036 |
|  | 1^st^ degree SMIBRD history | 0.39 (0.26) | .131 |
|  | Personal SMIBRD history | 0.48 (0.42) | .250 |
| Psychedelic use | No BIIP history | … | … |
|  | 2^nd^ degree BIIP history | 0.07 (0.28) | .809 |
|  | 1^st^ degree BIIP history | 0.18 (0.22) | .414 |
|  | Personal BIIP history | 0.07 (0.21) | .733 |
| Psychedelic use | No BRDDAMC history | … | … |
|  | 2^nd^ degree BRDDAMC history | 0.41 (0.43) | .347 |
|  | 1^st^ degree BRDDAMC history | 0.21 (0.52) | .686 |
|  | Personal BRDDAMC history | 1.16 (0.61) | .057 |
| Psychedelic use | No UBRD history | … | … |
|  | 2^nd^ degree UBRD history | -0.15 (0.24) | .519 |
|  | 1^st^ degree UBRD history | -0.27 (0.20) | .174 |
|  | Personal UBRD history | 0.50 (0.37) | .172 |
| Psychedelic use | No BPD history | … | … |
|  | 2^nd^ degree BPD history | 0.00 (0.42) | .998 |
|  | 1^st^ degree BPD history | -0.08 (0.25) | .764 |
|  | Personal BPD history | 0.27 (0.24) | .270 |
| Psychedelic use | No OCPD history | … | … |
|  | 2^nd^ degree OCPD history | -0.50 (0.39) | .205 |
|  | 1^st^ degree OCPD history | 0.21 (0.33) | .533 |
|  | Personal OCPD history | -0.19 (0.61) | .752 |
| Psychedelic use | No SCZPD history | … | … |
|  | 2^nd^ degree SCZPD history | 0.30 (0.42) | .485 |
|  | 1^st^ degree SCZPD history | 0.56 (0.44) | .199 |
|  | Personal SCZPD history | 0.13 (0.63) | .838 |
| Psychedelic use | No SCZAD history | … | … |
|  | 2^nd^ degree SCZAD history | 0.71 (0.59) | .231 |
|  | 1^st^ degree SCZAD history | 0.49 (0.39) | .218 |
|  | Personal SCZAD history | -0.47 (0.48) | .324 |
| Change in severity of perceptual abnormalities | | | |
| Predictor variable | Interaction term | β (SE) | p |
| Psychedelic use | No SMIBRD history | … | … |
|  | 2^nd^ degree SMIBRD history | 0.87 (0.39) | .027 |
|  | 1^st^ degree SMIBRD history | 0.39 (0.26) | .129 |
|  | Personal SMIBRD history | -0.81 (0.44) | .065 |
| Psychedelic use | No BIIP history | … | … |
|  | 2^nd^ degree BIIP history | -0.66 (0.28) | .016 |
|  | 1^st^ degree BIIP history | -0.43 (0.22) | .048 |
|  | Personal BIIP history | 0.31 (0.22) | .159 |
| Psychedelic use | No BRDDAMC history | … | … |
|  | 2^nd^ degree BRDDAMC history | 1.53 (0.44) | <.001 |
|  | 1^st^ degree BRDDAMC history | 0.47 (0.52) | .373 |
|  | Personal BRDDAMC history | -1.19 (0.62) | .056 |
| Psychedelic use | No UBRD history | … | … |
|  | 2^nd^ degree UBRD history | -0.19 (0.23) | .400 |
|  | 1^st^ degree UBRD history | -0.17 (0.20) | .391 |
|  | Personal UBRD history | 0.31 (0.38) | .417 |
| Psychedelic use | No BPD history | … | … |
|  | 2^nd^ degree BPD history | -2.09 (0.42) | <.001 |
|  | 1^st^ degree BPD history | -0.26 (0.25) | .303 |
|  | Personal BPD history | 0.18 (0.23) | .436 |
| Psychedelic use | No OCPD history | … | … |
|  | 2^nd^ degree OCPD history | -1.40 (0.39) | <.001 |
|  | 1^st^ degree OCPD history | -0.69 (0.33) | .038 |
|  | Personal OCPD history | 0.30 (0.63) | .635 |
| Psychedelic use | No SCZPD history | … | … |
|  | 2^nd^ degree SCZPD history | -0.79 (0.42) | .060 |
|  | 1^st^ degree SCZPD history | 0.85 (0.43) | .047 |
|  | Personal SCZPD history | -1.14 (0.67) | .087 |
| Psychedelic use | No SCZAD history | … | … |
|  | 2^nd^ degree SCZAD history | 2.14 (0.59) | <.001 |
|  | 1^st^ degree SCZAD history | -0.45 (0.39) | .250 |
|  | Personal SCZAD history | 1.61 (0.50) | .001 |
| Change in severity of bizarre experiences | | | |
| Predictor variable | Interaction term | β (SE) | p |
| Psychedelic use | No SMIBRD history | … | … |
|  | 2^nd^ degree SMIBRD history | 0.98 (0.39) | .011 |
|  | 1^st^ degree SMIBRD history | 0.24 (0.26) | .358 |
|  | Personal SMIBRD history | -1.01 (0.42) | .018 |
| Psychedelic use | No BIIP history | … | … |
|  | 2^nd^ degree BIIP history | -0.50 (0.28) | .070 |
|  | 1^st^ degree BIIP history | -0.19 (0.22) | .377 |
|  | Personal BIIP history | 0.06 (0.22) | .776 |
| Psychedelic use | No BRDDAMC history | … | … |
|  | 2^nd^ degree BRDDAMC history | -0.23 (0.44) | .604 |
|  | 1^st^ degree BRDDAMC history | 0.58 (0.52) | .262 |
|  | Personal BRDDAMC history | -1.23 (0.62) | .047 |
| Psychedelic use | No UBRD history | … | … |
|  | 2^nd^ degree UBRD history | -0.12 (0.23) | .613 |
|  | 1^st^ degree UBRD history | -0.30 (0.20) | .130 |
|  | Personal UBRD history | 0.17 (0.39) | .662 |
| Psychedelic use | No BPD history | … | … |
|  | 2^nd^ degree BPD history | -1.28 (0.42) | .211 |
|  | 1^st^ degree BPD history | -0.18 (0.25) | .468 |
|  | Personal BPD history | 0.17 (0.23) | .455 |
| Psychedelic use | No OCPD history | … | … |
|  | 2^nd^ degree OCPD history | -1.27 (0.40) | <.001 |
|  | 1^st^ degree OCPD history | -0.12 (0.33) | .727 |
|  | Personal OCPD history | -0.32 (0.63) | .612 |
| Psychedelic use | No SCZPD history | … | … |
|  | 2^nd^ degree SCZPD history | -1.27 (0.42) | .003 |
|  | 1^st^ degree SCZPD history | -0.67 (0.43) | .114 |
|  | Personal SCZPD history | -0.05 (0.65) | .939 |
| Psychedelic use | No SCZAD history | … | … |
|  | 2^nd^ degree SCZAD history | 0.89 (0.59) | .134 |
|  | 1^st^ degree SCZAD history | 0.02 (0.39) | .969 |
|  | Personal SCZAD history | -0.53 (0.50) | .290 |
| Change in severity of manic symptoms | | | |
| Predictor variable | Interaction term | β (SE) | p |
| Psychedelic use | No SMIBRD history | … | … |
|  | 2^nd^ degree SMIBRD history | -0.27 (0.39) | .485 |
|  | 1^st^ degree SMIBRD history | -0.33 (0.26) | .200 |
|  | Personal SMIBRD history | 0.25 (0.43) | .563 |
| Psychedelic use | No BIIP history | … | … |
|  | 2^nd^ degree BIIP history | -0.04 (0.28) | .874 |
|  | 1^st^ degree BIIP history | 0.31 (0.22) | .161 |
|  | Personal BIIP history | -0.08 (0.21) | .691 |
| Psychedelic use | No BRDDAMC history | … | … |
|  | 2^nd^ degree BRDDAMC history | -0.11 (0.44) | .798 |
|  | 1^st^ degree BRDDAMC history | 0.69 (0.52) | .179 |
|  | Personal BRDDAMC history | 0.63 (0.61) | .300 |
| Psychedelic use | No UBRD history | … | … |
|  | 2^nd^ degree UBRD history | -0.03 (0.23) | .885 |
|  | 1^st^ degree UBRD history | 0.11 (0.20) | .588 |
|  | Personal UBRD history | 0.06 (0.37) | .086 |
| Psychedelic use | No BPD history | … | … |
|  | 2^nd^ degree BPD history | -0.10 (0.42) | .804 |
|  | 1^st^ degree BPD history | 0.19 (0.25) | .457 |
|  | Personal BPD history | 0.07 (0.23) | .776 |
| Psychedelic use | No OCPD history | … | … |
|  | 2^nd^ degree OCPD history | 0.50 (0.40) | .212 |
|  | 1^st^ degree OCPD history | 0.00 (0.33) | .994 |
|  | Personal OCPD history | 0.02 (0.60) | .976 |
| Psychedelic use | No SCZPD history | … | … |
|  | 2^nd^ degree SCZPD history | 0.21 (0.42) | .622 |
|  | 1^st^ degree SCZPD history | 0.34 (0.43) | .427 |
|  | Personal SCZPD history | 1.57 (0.65) | .015 |
| Psychedelic use | No SCZAD history | … | … |
|  | 2^nd^ degree SCZAD history | 0.45 (0.59) | .448 |
|  | 1^st^ degree SCZAD history | 0.45 (0.40) | .255 |
|  | Personal SCZAD history | 0.67 (0.67) | .156 |
| This table shows interactions between psychedelic use and specific psychiatric histories on changes in the severity of psychotic or manic symptoms. β = standardized coefficient; SE = standard error; p = p-value; SMIBRD = substance/medication-induced bipolar and related disorder, BIIP = bipolar II disorder, BRDDAMC = bipolar and related disorder due to another medical condition, UBRD = unspecified bipolar and related disorder, BPD = borderline personality disorder, OCPD = obsessive-compulsive personality disorder, SCZPD = schizotypal personality disorder, SCZAD = schizoaffective disorder. The models controlled for age, gender identity, educational attainment, degree of religiosity, political affiliation, past two-month use of alcohol, nicotine products, cannabis products, MDMA, major stimulants, illicit narcotic analgesics/opioids, illicit benzodiazepines and barbiturates, inhalants, and other substances at follow-up, psychedelic use in the past two months at baseline, and survey year. Only specific diagnoses were included that had at least three responses in each cell among those who reported psychedelic use during the study period. | | | |

| **Table S9.** | | | |
| --- | --- | --- | --- |
| Change in severity of psychotic symptoms | | | |
| Predictor variable | Interaction term | β (SE) | p |
| Psychedelic use | … | 0.25 (0.05) | <.001 |
| Psychedelic use | No SZC history | … | … |
|  | 2^nd^ degree SCZ history | 0.02 (0.19) | .918 |
|  | 1^st^ degree SCZ history | 0.04 (0.24) | .870 |
|  | Personal SCZ history | -0.95 (0.52) | .069 |
| Psychedelic use | No BIP history | … | … |
|  | 2^nd^ degree BIP history | 0.39 (0.29) | .173 |
|  | 1^st^ degree BIP history | 0.11 (0.16) | .511 |
|  | Personal BIP history | -0.07 (0.23) | .763 |
| Change in severity of manic symptoms | | | |
| Predictor variable | Interaction term | *β* (SE) | *p* |
| Psychedelic use | … | 0.13 (0.05) | .017 |
| Psychedelic use | No SZC history | … | … |
|  | 2^nd^ degree SCZ history | 0.07 (0.19) | .713 |
|  | 1^st^ degree SCZ history | -0.03 (0.24) | .907 |
|  | Personal SCZ history | 1.32 (0.52) | .012 |
| Psychedelic use | No BIP history | … | … |
|  | 2^nd^ degree BIP history | 0.52 (0.29) | .068 |
|  | 1^st^ degree BIP history | -0.10 (0.16) | .546 |
|  | Personal BIP history | 0.73 (0.23) | .001 |
| This table shows (non-imputed) associations between psychedelic use and changes in severity of psychotic or manic symptoms, as well as interactions between psychedelic use and specific psychiatric histories (i.e., schizophrenia, bipolar I disorder) on changes in severity of psychotic or manic symptoms. β = standardized coefficient; SE = standard error; p = p-value; SCZ = schizophrenia, BIP = bipolar I disorder. The models controlled for age, gender identity, educational attainment, degree of religiosity, political affiliation, past two-month use of alcohol, nicotine products, cannabis products, MDMA, major stimulants, illicit narcotic analgesics/opioids, illicit benzodiazepines and barbiturates, inhalants, and other substances at follow-up, psychedelic use in the past two months at baseline, and survey year. | | | |
